# Lepr‐Expressing PDLSCs Contribute to Periodontal Homeostasis and Respond to Mechanical Force by Piezo1

**DOI:** 10.1002/advs.202303291

**Published:** 2023-08-08

**Authors:** Danting Zhang, Weimin Lin, Shuang Jiang, Peng Deng, Linfeng Liu, Qian Wang, Rui Sheng, Hui Sophie Shu, Lijun Wang, Weiguo Zou, Bo O. Zhou, Junjun Jing, Ling Ye, Bo Yu, Shiwen Zhang, Quan Yuan

**Affiliations:** ^1^ State Key Laboratory of Oral Diseases & National Clinical Research Center for Oral Diseases West China Hospital of Stomatology Sichuan University Chengdu 610041 China; ^2^ Division of Oral and Systemic Health Sciences School of Dentistry University of California Los Angeles Los Angeles CA 90095 USA; ^3^ State Key Laboratory of Cell Biology Shanghai Institute of Biochemistry and Cell Biology Center for Excellence in Molecular Cell Science Chinese Academy of Sciences University of Chinese Academy of Sciences Shanghai 200031 China; ^4^ Institute of Microsurgery on Extremities Shanghai Jiao Tong University Affiliated Sixth People's Hospital Shanghai 200233 China; ^5^ State Key Laboratory of Experimental Hematology Institute of Hematology & Blood Diseases Hospital Chinese Academy of Medical Sciences Tianjin 300020 China; ^6^ Department of Endodontics West China Hospital of Stomatology Sichuan University Chengdu 610041 China; ^7^ Division of Preventive and Restorative Sciences School of Dentistry University of California Los Angeles Los Angeles CA 90095 USA; ^8^ Department of Oral Implantology West China Hospital of Stomatology Sichuan University Chengdu 610041 China

**Keywords:** leptin receptor, lineage tracing, mechanical force, periodontal ligament stem cells (PDLSCs), piezo1

## Abstract

Periodontium supports teeth in a mechanically stimulated tissue environment, where heterogenous stem/progenitor populations contribute to periodontal homeostasis. In this study, Leptin receptor+ (Lepr+) cells are identified as a distinct periodontal ligament stem cell (PDLSC) population by single‐cell RNA sequencing and lineage tracing. These Lepr+ PDLSCs are located in the peri‐vascular niche, possessing multilineage potential and contributing to tissue repair in response to injury. Ablation of Lepr+ PDLSCs disrupts periodontal homeostasis. Hyper‐loading and unloading of occlusal forces modulate Lepr+ PDLSCs activation. Piezo1 is demonstrated that mediates the mechanosensing of Lepr+ PDLSCs by conditional *Piezo1*‐deficient mice. Meanwhile, Yoda1, a selective activator of Piezo1, significantly accelerates periodontal tissue growth via the induction of Lepr+ cells. In summary, Lepr marks a unique multipotent PDLSC population in vivo, to contribute toward periodontal homeostasis via Piezo1‐mediated mechanosensing.

## Graphical abstract

Periodontium supports teeth in a mechanically stimulated tissue environment with heterogenous stem/progenitor populations. Leptin receptor (Lepr) marks a unique multipotent periodontal ligament stem cell (PDLSC) population, which is activated by injury and mechanical stimulation. Piezo1 mediates the mechanosensing of Lepr+ PDLSCs to contribute toward periodontal homeostasis. This may carry significant clinical implications in future endeavors to develop functional periodontal regeneration.

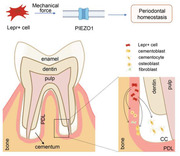

## 1 Introduction

The periodontium provides attachment and anchorage of teeth in the alveolar bone, while protecting underlying tissues against occlusal trauma.^[^ ^1^, ^2^ ^]^ The periodontal attachment, consisting of cementum covering roots of the teeth, periodontal ligament fibers, and the alveolar bone, share a common developmental origin in the neural crest.^[^ ^3^, ^4^, ^5^ ^]^ Functionally, the periodontium serves as an important connection and a shock absorbing medium by transmitting occlusal forces and mechanotransduction signals.^[^ ^6^ ^]^ Destruction of periodontium, through mechanical trauma or periodontal diseases, is a major cause of tooth loss in adults.^[^ ^7^ ^]^ Periodontitis is one of the most prevalent diseases, with an overall prevalence of 11% and affecting over 750 million worldwide. ^[^ ^8^ ^]^ Emerging evidence demonstrates a compelling connection between periodontitis with various systemic diseases such as diabetes, cardiovascular diseases and rheumatoid arthritis.^[^ ^9^, ^10^, ^11^, ^12^, ^13^ ^]^ Thus, understanding the mechanisms governing the homeostasis and pathology of periodontium has critical clinical implications for both oral and systemic health. The ultimate goal of periodontal therapy is to regenerate tooth‐supporting tissues to enable efficient mastication, and to maintain an oral immune barrier for systemic health.

Periodontal ligament stem cells (PDLSCs) play an important role in the development, maintenance, and regeneration of periodontal tissues. Through lineage tracing, several stem/progenitor cell populations in the periodontal ligament (PDL) space have been identified to contribute to the regeneration and repair of periodontium.^[^ ^14^, ^15^, ^16^, ^17^ ^]^ However, CD90+ cells are scarce in the adult periodontium during homeostasis.^[^ ^15^ ^]^ Gli1 marked nearly all the proliferative cells after 1‐week EdU assay,^[^ ^14^ ^]^ indicating that Gli1+ cells may constitute a heterogeneous population that include a subset of transit‐amplifying cells in addition to PDLSCs. Alpha‐smooth muscle actin(αSMA), another proposed marker of PDLSCs in vivo,^[^ ^16^ ^]^ was only traced for a relatively short time in the study. Considering the turnover of murine PDL cells is much slower than that of hair follicle,^[^ ^18^, ^19^, ^20^ ^]^ a longer tracing duration is needed to identify and verify the long‐term quiescent stem cells in the periodontium.

Leptin receptor (Lepr) has been identified as a marker of adult bone marrow stromal cells (BMSCs). Fate‐mapping analysis of Lepr‐expressing cells in the adult bone marrow suggested that Lepr+ BMSCs are the major source of osteoblasts and adipocytes.^[^ ^21^, ^22^ ^]^ Further research using *Lepr‐CreER;tdTomato* mice revealed that Lepr+ BMSCs give rise to most osteoblasts in adult limbs but few during development.^[^ ^23^ ^]^ Our group previously demonstrated that Lepr labels a population of postnatal alveolar skeletal stem cells (SSCs) essential for extraction socket healing and responding to PTH/PTH1R signaling.^[^ ^24^ ^]^ However, the role of Lepr+ cells in the periodontium remains unknown.

Mechanical stimuli regulate stem cell fate and behavior to guide developmental processes and maintain tissue homeostasis.^[^ ^25^ ^]^ During tissue regeneration, adult stem cells respond to mechanical stimuli and relay mechanotransduction signals to activate gene‐regulatory programs to promote stem cell differentiation.^[^ ^26^, ^27^ ^]^ The PDL tissues play an important role in distributing and transducing occlusal forces during normal mastication.^[^ ^28^ ^]^ In patients with occlusal disharmony and bruxism, which could be accounted in up to 31% of adults, hyper‐loading and unloading of occlusal forces lead to significant disruption of the periodontal homeostasis.^[^ ^29^ ^]^ Previous studies have confirmed that mechanical force regulates PDLSCs,^[^ ^30^, ^31^ ^]^ but the mechanism of PDLSCs in response to mechanical stimuli remains to be elucidated.

Here, we demonstrate that Lepr marks a distinct PDLSCs population that contributes to periodontium homeostasis and responds to mechanical force by Piezo1.

## 2 Results

### 2.1 Single‐Cell Atlas Reveals MSC‐Like Expression Pattern of *Lepr*

**Figure 1:**
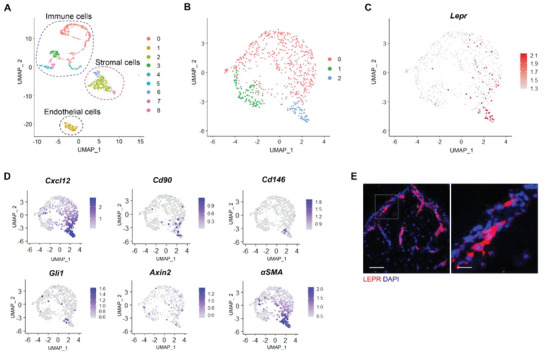
Single‐cell analysis reveals an MSC‐like expression pattern of *Lepr*. A) UMAP visualization of periodontium cells. Different cell populations were distinguished by color. B) UMAP plot representation of identified clusters in periodontium stromal cells. Cluster 0 represents fibroblasts expressing *Periostin* and *Col1a1*. Cluster 1 represents osteo‐lineage cells expressing *Dmp1* and *Sp7*. Cluster 2 represents the progenitor population that expresses classic stromal stem cell markers such as *Cxcl12*, *Cd90*, and *Cd146*. C) Distribution of *Lepr* in periodontium stromal cells. D) The expression of PDLSC marker genes projected onto UMAP atlas. E) LEPR immunofluorescent staining of adult mouse PDL cryostat section. Scale bar: left: 100 µm; right: 25 µm.

In order to understand the heterogeneous cell populations in periodontium, we first analyzed published single‐cell RNA sequencing (scRNA‐seq) data of periodontium around adult mouse molars. Three major cell types were identified, including stromal, endothelial, and immune cells (**Figure** **1**A). We categorized stromal cells into 3 clusters based on their differential gene expression patterns (Figure 1B). Cluster 0 expressed surface markers related to fibroblasts such as *Periostin* and *Col1a1*. Cluster 1 expressed osteo‐lineage markers *Dmp1* and *Sp7* (Figure S1A, Supporting Information). Cluster 2 represented the progenitor population that expressed classic stromal stem cell markers such as *Cxcl12, Cd90, and Cd146* (Figure 1C,D). Based on the gene expression dynamics, we constructed a pseudotime developmental tree. We found that the progenitor cluster had the lowest pseudotime value and therefore could be a developmental origin for other cell populations in the periodontium (Figure S1B, Supporting Information).

Interestingly, we found that *Lepr*, a well‐known marker for adult BMSCs, is highly expressed in the progenitor cluster, suggesting that Lepr may serve as a marker for a postnatal PDLSC population (Figure 1C). Compared to other PDLSC markers such as *Gli1* and *Axin2*, *Lepr* expression is higher and more specifically concentrated in the stem cells. In addition, *αSMA* and *Cxcl12* labeled too many fibroblasts besides PDLSCs (Figure 1D). Consistent with our transcriptome analysis, immunofluorescent staining verified that Lepr is expressed in mouse periodontium (Figure 1E).

### 2.2 Characterization of Lepr+ Cells During Development and Homeostasis of Periodontium

Next, we generated *Lepr‐CreER;tdTomato* mice to perform lineage tracing to examine the contribution of Lepr+ cells during periodontal morphogenesis. First, we injected tamoxifen at P5 and found that Lepr+ cells were present in the dental follicle two days after induction (Figure S2B,C, Supporting Information). After 23 days, the Lepr+ cells continued to contribute to PDL, alveolar bone, and cellular cementum (Figure S2C, Supporting Information). EdU administration revealed that Lepr+ cells actively proliferated during development (Figure S2D,E, Supporting Information).

**Figure 2:**
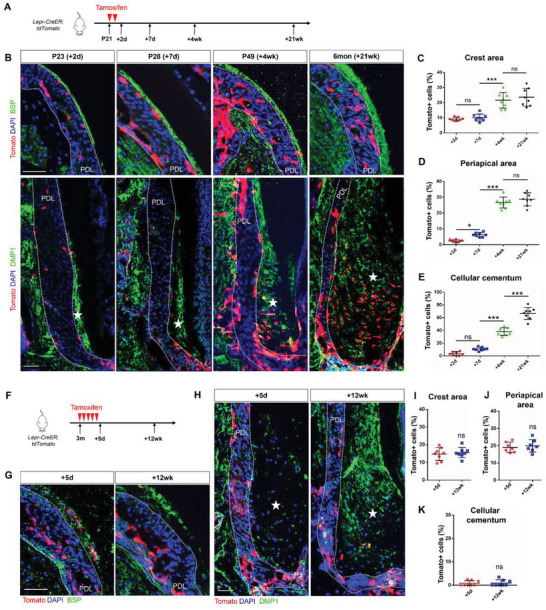
Lineage tracing of Lepr+ cells in periodontium. A) Schematics of tamoxifen induction from P21. B) Representative confocal images of the crest and periapical area of PDL from P21‐induced *Lepr‐CreER;tdTomato* mice. Scale bar: 50 µm. Pentagram: cellular cementum. C,D) Percentage of Tomato+/DAPI+ cells at the crest C) and periapical area D) of PDL from P21‐induced *Lepr‐CreER;tdTomato* mice. (*n* = 8). E) Percentage of Tomato+ /DAPI+ cells in cellular cementum from P21‐induced *Lepr‐CreER;tdTomato* mice (*n* = 8). F) Schematics of tamoxifen induction from 3 months of age. G,H) Images of the crest and periapical area of PDL from 3 months‐induced *Lepr‐CreER;tdTomato* mice. Scale bar: 50 µm. Pentagram: cellular cementum. I,J) Percentage of Tomato+/DAPI+ cells at the crest I) and periapical area J) of PDL from 3 months‐induced *Lepr‐CreER;tdTomato* mice. (*n* = 8). K) Percentage of Tomato+/DAPI+ cells in cellular cementum from 3 months‐induced *Lepr‐CreER;tdTomato* mice (*n* = 8). Data are presented as mean ± standard deviation. C–E) *P < 0.05, ***P<0.001; ns, not significant as determined by ANOVA. I–K) ns, not significant as determined by two‐tailed Student *t* tests.

Subsequently, we administered tamoxifen to adolescent *Lepr‐CreER;tdTomato* mice at P21 when PDL development completes and cementogenesis begins (**Figure** **2**A, Figure S2A, Supporting Information). Only a few Lepr+ cells were present in the periapical (2.39%) and alveolar crest area (9.14%) of PDL space two days after tamoxifen induction. The number of Lepr+ cells more than doubled in the periapical area (6.31%) one week after induction. Abundant progenies of Lepr+ cells were observed both in the crest area (21.60%) and the periapical area (26.66%) within PDL space one month after induction. The number of Lepr+ cells in the periodontium plateaued by the age of 6 months (Figure 2B–D). Notably, as cellular cementum started to develop from P21 to 6 months of age, we observed a significant increase in Lepr+ cementocytes for up to 66.85% of the cementocytes at 6 months (Figure 2B,E).

To investigate whether the adult Lepr+ cells persists in periodontium, we injected tamoxifen to 3‐month‐old *Lepr‐CreER;tdTomato* mice for 5 consecutive days (Figure 2F). After a 3‐month chase, there was no significant change in the number of Lepr+ cells within the PDL space (Figure 2G–J). Interestingly, Lepr+ cells rarely contributed to cementocytes after 3 months (Figure 2K).

### 2.3 Lepr+ Cells Exhibit Biological Characteristics of Stem Cells

**Figure 3:**
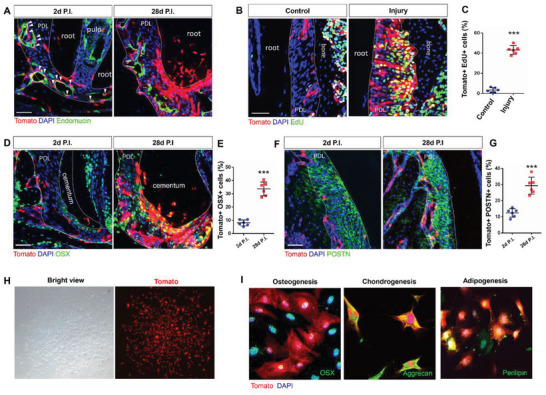
Characteristics of Lepr+ PDLSCs in vivo and in vitro. A) Endomucin staining was performed on *Lepr‐CreER;tdTomato* mice which induced 2 days from P21. P.I., post‐induction. Arrows indicate the Tomato+ cells located in the peri‐vascular niche. Scale bar: 50 µm. B,C) Representative images and percentage of Tomato+EdU+/Tomato+ cells from adult *Lepr‐CreER;tdTomato* mice with PDL injury and control group (*n* = 6). Scale bar: 50 µm. D–G) Osterix D,E) and Periostin F,G) staining and statistical analysis of *Lepr‐CreER;tdTomato* mice which induced 2 days from P21 (*n* = 6). P.I., post‐induction. Scale bar: 50 µm. H) Colony formation from single Tomato+ cell. I) Osterix, Aggrecan, and Perilipin staining indicated the osteogenesis(left), chondrogenesis(middle), and adipogenesis(right) potential of Lepr+ cells. Data are presented as mean ± standard deviation. ***P<0.001 as determined by two‐tailed Student *t*‐tests.

Peri‐vascular niches are known to provide a ubiquitous reserve of multilineage stem cells.^[^ ^32^ ^]^ To further investigate the location of Lepr+ cells in vivo, we performed Endomucin staining to visualize the vasculature and found that Lepr+ cells were located in close proximity to the blood vessels, then migrated out of the peri‐vascular niche to contribute to the formation of the periodontium (**Figure** **3**A). In order to investigate whether Lepr+ cells contribute to tissue repair in response to injury, a consecutive EdU administration was performed on the adult *Lepr‐CreER;tdTomato* mice with PDL injury. Under physiological conditions, Lepr+ cells within PDL stayed in a quiescent state with minimal proliferative activity. However, Lepr+ cell proliferation was markedly elevated in response to PDL injury (Figure 3B,C).

PDLSCs could differentiate into fibroblasts, cementoblasts, and osteoblasts in the periodontium. To examine the multilineage potentials of Lepr+ cells in vivo, we induced adolescent *Lepr‐CreER;tdTomato* mice and found that on day 2, majority of Lepr+ cells were negative for both Osterix (OSX), an osteoblast marker, and Periostin (POSTN), a marker for periosteum fibroblasts (Figure 3D–G). After a 4‐week chase, 33.70% Lepr+ cells were OSX positive and differentiated to osteoblasts (lining bone surface and located within PDL space), cementoblasts (lining cementum surface) and cementocytes (embedded in mineralized cementum matrix) (Figure 3D,E). Meanwhile, ≈29.32% of Lepr+ cells differentiated to fibroblasts during adolescence (Figure 3F,G). In addition, immunofluorescent staining showed that Lepr+ cells partially expressed classical MSC markers (Figure S4A,B, Supporting Information), suggesting that Lepr marks a distinct subset of PDLSCs.

To verify the migration of Lepr+ cells, we also carried out the clonal assay with low‐dose tamoxifen administration in vivo. *Lepr‐CreER;tdTomato* mice were induced with low and full tamoxifen dose at the age of P21. Compared to the full‐dose group, fewer Lepr+ cells were activated and individual Lepr+ cells were detected within the PDL space two days after low‐dose induction (Figure S3A,Bi, Supporting Information). On day 21, isolated clones were identified (Figure S3B ii,iii, Supporting Information) and these encompassed the progenies which migrated to the cementum and differentiated into cementoblasts (Figure S3B iv, Supporting Information), as well as the progenies which migrated to the alveolar bone and differentiated into osteoblasts (Figure S3B v, Supporting Information).

Next, we isolated PDLSCs from the molars of adult *Lepr‐CreER;tdTomato* mice to verify the stemness of Lepr+ cells in vitro. Hematopoietic and immune cell markers including CD31 and CD45 were not detectable (Figure S4C, Supporting Information), while stromal cell markers CD90, CD146, and CD105 were highly expressed in cultured PDLSCs (Figure S4D, Supporting Information). We observed that a single Lepr+ cell was able to proliferate and form a CFU‐F colony (Figure 3H). Subsequently, the overlapped staining of Osterix, Aggrecan, and Perilipin with Tomato confirmed the multilineage potency of the Lepr+ PDLSCs passaging from the colony to differentiate into osteoblasts, chondrocytes, and adipocytes, *respectively* (Figure 3I).

### 2.4 Ablation of Lepr+ PDLSCs Disrupts Periodontium Homeostasis

**Figure 4:**
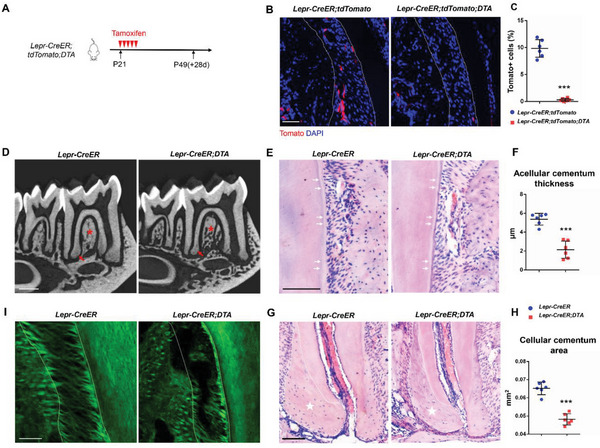
Ablation of Lepr+ PDLSCs disrupts periodontium homeostasis. A) Schematics of tamoxifen induction. B,C) Representative images and percentage of Tomato+ cells in PDL from *Lepr‐CreER;tdTomato;DTA* and control mice (*n* = 6). Scale bar: 50 µm. D) Representative µCT images of mandible in *Lepr‐CreER;DTA* and control mice. Scale bar: 500 µm. Arrows indicate the cellular cementum. Asterisks indicate the alveolar bone. E–H) H&E staining and statistical analysis of the acellular E,F) and cellular G,H) cementum in *Lepr‐CreER;DTA* and control mice (*n* = 6). Scale bar: 100 µm. Arrows E) indicate the acellular cementum. Dotted lines G) outline ROI of the cellular cementum. I) SHG microscopy of PDL fibrils in *Lepr‐CreER;DTA* and control mice. Scale bar: 50 µm. Data are presented as mean ± standard deviation. ***P<0.001 as determined by two‐tailed Student *t* tests.

To further explore the role of Lepr+ PDLSCs in periodontal homeostasis, we performed the cell ablation assay using *Lepr‐CreER;tdTomato;DTA* mice with tamoxifen administered since P21 (**Figure** **4**A). We found that 96.8% of Lepr+ cells were depleted in *Lepr‐CreER;tdTomato;DTA* mice after induction (Figure 4B,C). Micro–computed tomography (µCT) images revealed a cementum hypoplasia and alveolar bone destruction 28 days after ablation (Figure 4D). H&E staining further confirmed that the extracellular matrix (ECM) mass in the Lepr‐ablated acellular and cellular cementum was notably reduced (Figure 4E–H). Second harmonic generation (SHG) microscopy showed sparse and disoriented fibrils in Lepr‐ablated PDL compared to the control (Figure 4I). Additionally, ablation of Lepr+ cells in adult DTA mice (Figure S5A,B, Supporting Information) also showed significant cementum hypoplasia and alveolar bone destruction (Figure S5C–E, Supporting Information), as well as sparse fibrils in Lepr‐ablated PDL space (Figure S5F, Supporting Information). Autophagy and apoptosis significantly impact stem cell maintenance, and might contribute to the disruption of periodontium with Lepr ablation.^[^ ^33^, ^34^ ^]^ However, there was minimal autophagy marker P62 signal detected in the PDL space in Lepr‐ablated and control mice (Figure S6A, Supporting Information). Few TUNEL+ apoptotic cells were detected in both Lepr‐ablated and control group (Figure S6B, Supporting Information), suggesting apoptosis and autophagy are not responsible for the disruption of PDL homeostasis after ablation of Lepr+ cells.

### 2.5 Lepr+ PDLSCs are Activated by Occlusal Forces

**Figure 5:**
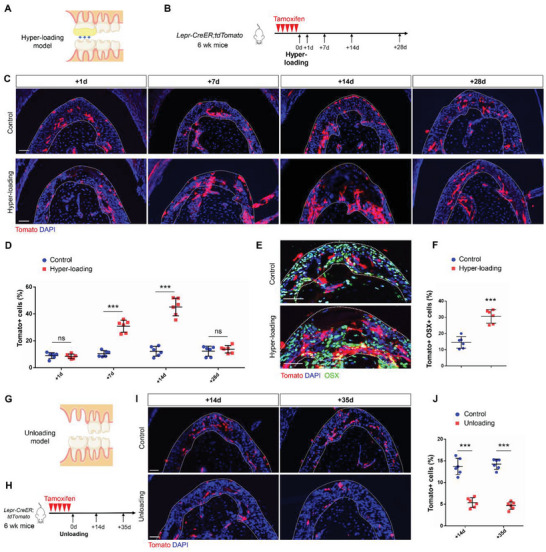
Lepr+ PDLSCs are activated by occlusal forces. A,B) Schematics of hyper‐loading and tamoxifen administration of *Lepr‐CreER;tdTomato* mice. C,D) Representative images and percentage of Tomato+ cells in PDL on 1d, 7d, 14d, 28d after hyper‐loading (*n* = 6). Scale bar: 50 µm. E,F) Osterix staining and percentage of Tomato+ OSX+/ Tomato+ cells. (*n* = 6). Scale bar: 50 µm. G,H) Schematics of unloading and tamoxifen administration of *Lepr‐CreER;tdTomato* mice. I,J) Representative images and percentage of Tomato+ cells in PDL on 14d and 35d after unloading (*n* = 6). Scale bar: 50 µm. Data are presented as mean ± standard deviation. D,J) ***P<0.001; ns, not significant as determined by ANOVA. F) ***P<0.001 as determined by two‐tailed Student *t*‐tests.

Mechanical stress guides stem cell behavior in development and homeostasis.^[^ ^25^ ^]^ In order to investigate the impact of occlusal forces on Lepr+ PDLSCs, we generated an occlusal force hyper‐loading model on the left maxillary first molar of *Lepr‐CreER;tdTomato* mice (**Figure** **5**A,B). On days 7 and 14, hyper‐loading stimulated an increase of Lepr+ cells. Over time, the number of Lepr+ cells in the hyper‐loading group gradually decreased to a similar level compared to the control group (Figure 5C,D). Increased osteogenic staining suggested that hyper‐loading promotes osteogenic differentiation of Lepr+ cells (Figure 5E,F; Figure S7A,B, Supporting Information).

In addition, we extracted the upper left molars to unload occlusal forces on the opposing lower left molars (Figure 5G,H). Lepr+ cells were significantly reduced compared to the control group at 14d and 35d after unloading (Figure 5I,J). Meanwhile, lack of mechanical stress results in impaired osteogenic differentiation of Lepr+ cells (Figure S7C,D, Supporting Information), suggesting that physiological mechanical force is required for the activation of Lepr+ PDLSCs.

### 2.6 Piezo1 is Essential for Lepr+ PDLSCs to Respond to Mechanical Force

**Figure 6:**
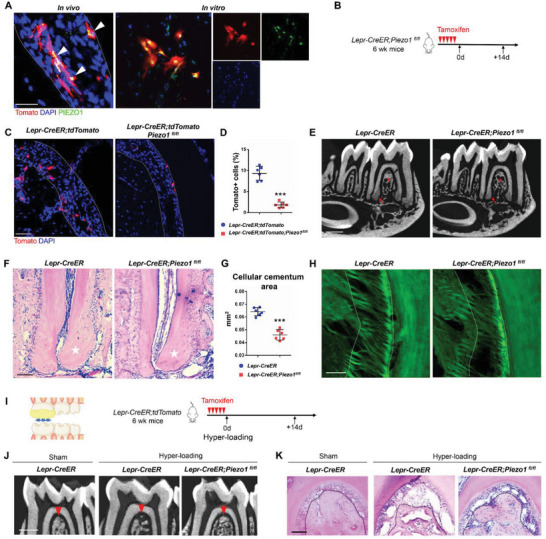
Lepr+ PDLSCs respond to mechanical force via Piezo1. A) PDL cryostat section (left) and cultured PDLSCs (right) of *Lepr‐CreER;tdTomato* mice with PIEZO1 staining. Scale bar: 25 µm. B) Schematics of tamoxifen induction in *Lepr‐CreER;Piezo1^fl/fl^* and control mice. C,D) Representative images and percentage of Tomato+ cells in PDL from *Lepr‐CreER;tdTomato;Piezo1^fl/fl^* and control mice (*n* = 6). Scale bar: 50 µm. E) Representative µCT images of mandible in *Lepr‐CreER;Piezo1^fl/fl^* and control mice. Scale bar: 500 µm. Arrows indicate the cellular cementum. Asterisks indicate the alveolar bone. F,G) H&E staining and statistical analysis of the cellular cementum in *Lepr‐CreER;Piezo1^fl/fl^* and control mice (*n* = 6). Scale bar: 100 µm. Dotted lines outline ROI of the cellular cementum. H) SHG microscopy of PDL fibrils in *Lepr‐CreER;Piezo1^fl/fl^* and control mice. Scale bar: 50 µm. I) Schematics of hyper‐loading and tamoxifen administration in *Lepr‐CreER;Piezo1^fl/fl^* and control mice. J,K) Representative µCT images J) and H&E staining K) of mandible in *Lepr‐CreER;Piezo1^fl/fl^* and control mice. Scale bar: J) 500 µm; K) 100µm. Arrows indicate the alveolar crest J). Dotted lines outline the crest K). Data are presented as mean ± standard deviation. ***P<0.001 as determined by two‐tailed Student *t* tests.

Piezo1 is an essential component of mechanically activated cation channels in vertebrates, which is widely involved in mechano‐transduction.^[^ ^35^ ^]^ Immunofluorescence staining confirmed that Piezo1 is expressed in Lepr+ PDLSCs both in vivo and in vitro (**Figure** **6**A). Next, we generated *Lepr‐CreER;tdTomato;Piezo1^fl/fl^* mice to delete *Piezo1* in Lepr+ cells (Figure 6B). Notably, the number of Lepr+ cells was significantly reduced after *Piezo1* deletion (Figure 6C,D), suggesting that Piezo1 signaling is important for the maintenance of Lepr+ cells. µCT images showed the cellular cementum formation and alveolar bone mass were reduced in *Piezo1*‐deficient mice (Figure 6E), while the ECM mass of cementum in *Piezo1*‐deficient mice was also markedly lower (Figure 6F,G). SHG images revealed sparse and disorganized fibrils after *Piezo1* deletion compared to the highly organized PDL collagen fibrils in control mice (Figure 6H). Taken together, Piezo1 is essential for the contribution of Lepr+ PDLSCs for the homeostasis of periodontium.

In order to investigate the impact of Piezo1 for Lepr+ PDLSCs to sense force, we performed the hyper‐loading model on *Piezo1*‐deficient mice (Figure 6I). µCT images and histological experiments showed that *Piezo1*‐deficient mice were resistant to periodontium destruction induced by hyper‐loading (Figure 6J,K), suggesting that Piezo1 is essential for Lepr+ PDLSCs to respond to mechanical force.

### 2.7 Yoda1 Administration Accelerates Periodontal Tissue Growth

**Figure 7:**
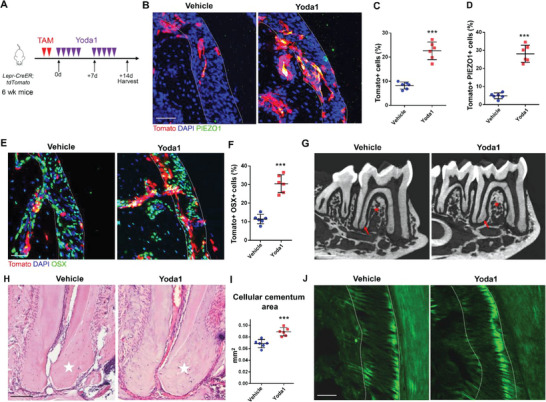
Yoda1 accelerates periodontal tissue growth. A) Schematics of tamoxifen induction and Yoda1 administration of *Lepr‐CreER;tdTomato* mice. B) Representative images of PIEZO1 staining in PDL of Yoda1 and vehicle group. Scale bar: 50 µm. C) Percentage of Tomato+ cells in PDL of Yoda1 and vehicle group (*n* = 6). D) Percentage of Tomato+ PIEZO1+/Tomato+ cells in PDL of Yoda1 and vehicle group (*n* = 6). E,F) Representative Osterix staining images and percentage of Tomato+ OSX+ /Tomato+ cells in PDL of Yoda1 and vehicle group (*n* = 6). Scale bar: 50 µm. G) Representative µCT images of mandible in Yoda1 and vehicle group. Scale bar: 500 µm. Arrows indicate the cellular cementum; asterisks indicate the alveolar bone. H,I) H&E staining and statistical analysis of the cellular cementum in Yoda1 and vehicle group (*n* = 6). Scale bar: 100 µm. Dotted lines outline ROI of the cellular cementum. J) SHG microscopy of PDL fibrils in Yoda1 and vehicle group. Scale bar: 50 µm. Data are presented as mean ± standard deviation. ***P<0.001 as determined by two‐tailed Student *t*‐tests.

Next, we sought to investigate the impact of activating Piezo1 on periodontium by administrating Yoda1, a pharmacological agonist of Piezo1, on *Lepr‐CreER;tdTomato* mice (**Figure** **7**A).^[^ ^36^ ^]^ More Lepr+ cells were detected within the PDL after 14 days of Yoda1 administration (Figure 7B,C). In addition, the percentage of Lepr+PIEZO1+/Lepr+ cells increased compared with control group (Figure 7B,D). OSX staining revealed Yoda1‐ induced osteogenic differentiation of Lepr+ PDLSCs (Figure 7E,F). Consistently, administration of Yoda1 significantly increased the cementum formation and alveolar bone mass as evidenced by µCT (Figure 7G), as well as increased ECM mass of cellular cementum (Figure 7H,I). Compared to non‐treated controls, a denser organization of fibrils was observed in the PDL space in Yoda1‐treated mice (Figure 7J), implicating that activation of Piezo1 could activate Lepr+ PDLSCs to promote periodontal regeneration.

## 3 Discussion

Distinct stem cell populations contribute to the complex soft and hard tissues of periodontium to maintain its functions in tooth anchoring and force transduction.^[^ ^37^ ^]^ Presently, PDLSCs were regarded as a promising tool for MSC‐based tissue engineering in regeneration therapy, due to their accessibility, self‐renewal and immunomodulatory properties comparable to BMSC.^[^ ^38^ ^]^ Previous lineage‐tracing studies have identified multiple stem/progenitor cell markers in the periodontium, such as αSMA and Axin2.^[^ ^4^, ^14^, ^16^, ^39^, ^40^ ^]^ Recently, Men et al. identified Gli1 as an adult PDLSCs marker, and sclerostin from the osteocyte is a negative feedback regulator for Gli1+ PDLSCs activity.^[^ ^14^ ^]^ They performed Lepr immunostaining on tamoxifen‐induced adult *Gli1‐Cre^ERT2^;Ai14* mice and found that Gli1+ PDL cells did not overlap with Lepr+ cells two days after tamoxifen induction. Interestingly however, sixty days after induction, Lepr+ cells were positively labeled as Gli1 lineage, indicating that the Lepr+ cells may mark a distinct developmental subpopulation that eventually converged with Gli1+ cells.^[^ ^14^ ^]^ Moreover, considering that Gli1 marked nearly all the proliferative cells after 1‐week EdU assay,^[^ ^14^ ^]^ Gli1 may label a heterogeneous population that included a subset of transit‐amplifying cells in addition to PDLSCs. In this study, we found that Lepr labels a peri‐vascular quiescent PDLSCs population, possessing stem cell characteristics, such as actively proliferating in injury, differentiating into osteoblasts, cementoblasts, and fibroblasts in vivo and undergoing multilineage differentiation in vitro (Figure 3), thus Lepr could present as a promising tool to identify and isolate critical PDLSCs for periodontal regeneration.

Developmentally, the periodontium is derived from the neural crest, which develops into various structures including craniofacial bones and cartilage, teeth, and cranial ganglia.^[^ ^3^ ^]^ Previous studies have shown that Lepr+ cells contribute to the development and regeneration of the cranial bone. ^[^ ^41^ ^]^ ScRNA‐seq analysis showed that Lepr is expressed in the human jawbone periosteal stem cells.^[^ ^42^ ^]^ In addition, our previous study has shown that alveolar bone contains a tissue‐resident quiescent Lepr+ SSCs population which was rapidly activated in response to injury.^[^ ^24^ ^]^ However, previous studies investigating the role of Lepr+ cells in craniofacial structures were all carried out with *Lepr‐Cre* mice,^[^ ^14^, ^24^, ^41^ ^]^ where Lepr marks all the offspring cells. Utilizing the inducible *Lepr‐CreER* system, in which the Cre activation is controlled at the precise time when tamoxifen is injected, we first unveil that Lepr marks a distinct PDLSCs population contributing to periodontium homeostasis. Furthermore, given that PDL cells appear to be a potential source of LepR+ cells in the socket healing,^[^ ^39^ ^]^ and that Lepr+ PDLSCs can also give rise to osteoblasts in vivo, they likely participate in bone regeneration of tooth socket as well.

Recently, scRNA‐seq‐based findings revealed the cellular heterogeneity in periodontium and their developmental precursor cells in the dental follicle.^[^ ^15^, ^37^, ^43^, ^44^, ^45^ ^]^ These single‐cell atlas revealed previously unidentified dental cell types and their hierarchical relationships. Zhao et al. found that Axin2+ and CD90+ cells formed distinct precursor populations of cementoblasts: Axin2 maintained expression in cementoblasts, whereas CD90+ cells almost vanished in the adult periodontium.^[^ ^15^ ^]^ Our scRNA‐seq analysis revealed that PDL Lepr exhibited MSC‐like expression pattern (Figure 1B,C). Notably, Lepr expression, as compared to Axin2, was higher and more specifically concentrated in the PDLSC population (Figure 1C,D).

Proper masticatory loading is required for periodontal homeostasis.^[^ ^29^ ^]^ Hyper‐loading of the dentition causes PDL widening and bone loss, ^[^ ^46^ ^]^ while losing occlusal forces results in atrophy of periodontal tissues.^[^ ^47^, ^48^ ^]^ Mechanical forces are transduced in stem cell niches to regulate cell fate and biological behavior, and to guide their self‐renewal and differentiation processes.^[^ ^25^ ^]^ PDLSCs are constantly regulated by mechanical force stimuli and contribute to the mechanosensing of periodontium.^[^ ^49^, ^50^ ^]^ Men et al. showed that physiological occlusal forces regulate the activation of Gli1+ PDLSCs.^[^ ^14^ ^]^ Similarly, the proliferation of Wnt‐responsive progenitor cells in PDL was triggered by masticatory loading.^[^ ^51^, ^52^ ^]^ Consistent with previous studies, we demonstrated that physiologic mechanical force is essential for the maintenance of Lepr+ PDLSCs.

Piezo1, a mechanosensitive ion channel, has been proposed to mediate innate immunity, bone remodeling, and cell fate determination under the physiological and pathological circumstances.^[^ ^53^, ^54^, ^55^ ^]^ Wang et al. identify Piezo1 as a regulator that coordinates the osteoblast–osteoclast crosstalk.^[^ ^56^ ^]^ During orthodontic tooth movement, Piezo1 channel was activated in PDL cells and mediated the osteogenic and osteoclastic activities.^[^ ^57^ ^]^ Recently, *Piezo1* in Osteolectin+ cells, a subset of transit‐amplifying osteogenic progenitors of Lepr+ cells, was demonstrated to maintain a peri‐arteriolar niche for osteogenic and lymphoid progenitors in femur.^[^ ^58^ ^]^ In that study, the deletion of *Piezo1* in Lepr+ cells did not significantly reduce femur bone mineral density. However, we found obviously reduced ECM mass of cementum in *Lepr‐CreER;Piezo1^fl/fl^* mice. This may be due to the distinct niche environments and stem cell features between the PDL and long bone.^[^ ^59^, ^60^ ^]^

Yoda1, the pharmacological agonist of Piezo1 has been used in the study of the cardiovascular system, bone homeostasis, iron metabolism, and cancer therapy since 2015.^[^ ^58^, ^61^, ^62^, ^63^ ^]^ In this study, we found that Yoda1 significantly accelerated periodontal tissues growth via activation of Lepr+ cells (Figure 7), suggesting that Yoda1 may be an effective therapeutic target to accelerate periodontal repair and regeneration.

In summary, we demonstrated that Lepr marked a distinct PDLSCs population exhibiting multilineage potentials. Lepr+ cells were sustained as a quiescent population but could be activated by injury and mechanical stimulation. Mechanistically, Piezo1‐mediated mechanosensing in the periodontium represents a novel aspect of the interplay of stem cells with the environmental cues and carries significant clinical implications in future endeavors to develop functional periodontal regeneration.

## 4 Experimental Section

### Animals

*Lepr‐CreER* and *ROSA‐DTA* mice were kindly provided by Bo O. Zhou (University of Chinese Academy of Sciences, Shanghai). *Piezo1* strain is the gift from Weiguo Zou (University of Chinese Academy of Sciences, Shanghai). *ROSA‐tdTomato* (007905) mice were purchased from Jackson Laboratory. All procedures were approved by the Subcommittee on Research and Animal Care (SRAC) of Sichuan University (approval number: WCHSIRB‐D‐2021‐041). All mice were under a specific pathogen‐free environment and analyzed regardless of sex. To induce Cre recombinase activity, unless otherwise specified, *Lepr‐CreER* mice were intraperitoneally injected with 100 µL tamoxifen (10 mg mL^−1^) dissolved in corn oil for 5 consecutive days. For long duration EdU assay, mice received intraperitoneally EdU (Sigma‐Aldrich) injection (200 mg kg^−1^) at 12‐hr interval for one week. For Yoda1 administration, mice were injected Yoda1 (Sigma‐Aldrich) intraperitoneally (5 mmol kg^−1^) following the previously published protocol.^[^ ^64^ ^]^

### Surgeries

The Mice PDL injury model was created on 6‐week‐old *Lepr‐CreER;tdTomato* mice which were induced tamoxifen for 5 days before. After mice were anesthetized with ketamine (100 mg kg^−1^) and xylazine (10 mg kg^−1^), the left mandibular first molars were swang side‐to‐side to tear the periodontal ligament fibers by ophthalmic forceps. Occlusal force hyper‐loading and unloading models were generated on 6‐week‐old *Lepr‐CreER;tdTomato* mice which were induced with tamoxifen for 5 days before. For the hyper‐loading model, composite resin adhered to the occlusal surface of left maxillary first molars under anesthesia. The retention of resin was checked one week after surgery and re‐adhered if the resin missing. For the unloading model, left maxillary first molars were extracted with 26G syringe needles and forceps. After the surgery, mice were housed individually. The left mandibles were harvested and analyzed.

### Histological Preparation and Staining

The mandibles were fixed by cardiac perfusion and then stored in 4% paraformaldehyde (PFA) for 1 day followed by 2‐week decalcification in 20% EDTA (pH 7.4) at 4 °C. For immunofluorescence, OCT‐embedded mandibles were cut into 15‐µm‐thick sections using a freezing microtome (Leica CM3050 S). The staining was performed as previously described.^[^ ^65^ ^]^ Sections were incubated overnight at 4 °C with primary antibodies: goat‐anti‐LEPR(1:200, BAF497, R&D), rabbit‐anti‐BSP (1:200,ab270605, Abcam), sheep‐anti‐DMP1 (1:200, AF4386, R&D), rat‐anti‐Endomucin(1:200, sc‐65495, Santa cruz), mouse‐anti‐CD90(1:200, sc‐53116 Santa cruz), mouse‐anti‐CD105(1:200, sc‐18838, Santa cruz), mouse‐anti‐CD146(1:200, sc‐18837, Santa cruz), mouse‐anti‐CD31(1:200, sc‐376764, Santa cruz), rat‐anti‐CD45(1:200, sc‐19597, Santa cruz), rabbit‐anti‐αSMA(1:400, ab184705, Abcam), rabbit‐anti‐Osterix(1:300, ab22552, Abcam), rabbit‐anti‐perilipin(1:800, ab3526, Abcam), mouse‐anti‐Aggrecan(1:200, sc‐166951, Santa cruz), rabbit‐anti‐PIEZO1(1:200, NBP1‐78446, Novus). Donkey anti‐goat Alexa Fluor 647(1:200, ab150131, Abcam), donkey anti‐rabbit Alexa Fluor 647 (1:200, ab150067, Abcam), donkey anti‐mouse Alexa Fluor 647(1:200, ab150107, Abcam), donkey anti‐rat Alexa Fluor 647(1:200, ab150155, Abcam), donkey anti‐sheep Alexa Fluor 647(1:200, ab150179, Abcam) were used as secondary antibodies. DAPI (D8417, Sigma) was used for counterstaining. The images were acquired with a laser scanning confocal microscopy (LSCM; Olympus FV3000). For H&E staining and SHG microscopy, decalcified samples were dehydrated in graded ethanol and embedded in paraffin. Mandibles were sectioned into 5‐µm slices using the microtome (Leica RM2255). H&E staining was performed according to the manufacturer's instruction (Biosharp). SHG microscopy was performed with a multiphoton microscope (Leica SP8 DIVE).

### PDLSCs Culture and In Vitro Assays

PDLSCs were isolated and cultured following the previously published method.^[^ ^14^ ^]^ Briefly, mandibles of adult *Lepr‐CreER;tdTomato* mice with 5 days of tamoxifen induction were dissected after anesthetization. Muscles and gingival tissue were removed. Then the molars were extracted and verified the integrity of root under microscopy. Teeth explants were placed on a culture dish with a digestion medium. The isolated PDL cells were cultured with a stem cell expansion medium. Osteogenic, chondrogenic, and adipogenic differentiation induction were performed as the previously published method and detected by immunofluorescent staining with Osterix, Aggrecan, and Perilipin, *respectively*.^[^ ^14^ ^]^

### µCT Image

µCT was performed as previously described.^[^ ^66^, ^67^ ^]^ The mandibles were fixed in 4% PFA for 1 day at 4 °C, then they were stored in phosphate‐buffered saline (PBS) at 4 °C until scanned with µCT–50 system (Scanco Medical) at a voxel size of 5 µm. IMARIS software (Version 9.1.2) was used for image processing.

### Analysis of scRNA‐seq Data

The scRNA‐seq data were obtained from the GEO database (GSE160358).^[^ ^15^ ^]^ To perform the scRNA‐seq analysis, the gene expression matrix was downloaded and then loaded into the Seurat package (v4.1). For quality control, genes expressed in less than 3 cells, mitochondrial genes higher than 10%, and cells with less than 200 expressed genes were filtered out. The principal component analysis (PCA) was performed. The cells were divided into several subclusters at resolution = 1, then the Uniform Manifold Approximation and Projection (UMAP) dimensionality reduction (dims = 1:30) was performed based on PCA results. The expression of several classical marker genes was displayed in the UMAP plot. Then, the clusters identified as PDLSCs, osteo‐lineage cells, and fibroblasts were extracted with subset function and converted into “Monocle” format. Pseudotime analysis was performed to infer the differentiation trajectories of different cell clusters through Monocle2 package.

### Statistical Analyses

All data were represented as mean ± standard deviation. Statistical difference was analyzed by unpaired two‐tailed Student's *t*‐test for two independent groups (When variances are unequal between groups, Welch's t‐test was applied). One‐way ANOVA followed by Tukey's post‐hoc test was used for multiple comparisons. All data were normally distributed. GraphPad Prism was used for all analyses and *P* < 0.05 was considered as significant difference.

## Conflict of Interest

The authors declare no conflict of interest.

## Author Contributions

D.Z conceived, designed, performed most of the experiments, and wrote the draft. W.L., S.J., L.L., Q.W., R.S., H.S., and L.W. contributed to data acquisition and analysis. P.D., W.Z., B.O.Z., J.J., and L.Y. contributed to data analysis and revised the draft. B.Y. provided valuable comments, helped to analyze the data, and revised the draft. S.Z. and Q.Y. conceived, designed the research, and revised the draft. All authors gave final approval and agreed to be accountable for all aspects of the work.

## Supporting information

Supporting Information

## Data Availability

The data that support the findings of this study are available from the corresponding author upon resonable request.
