# Supplementary material for: Lepr‐Expressing PDLSCs Contribute to Periodontal Homeostasis and Respond to Mechanical Force by Piezo1
Source: Adv Sci (Weinh). 2023 Aug 8;10(29):2303291. doi: 10.1002/advs.202303291 (PMC10582421; doi:10.1002/advs.202303291)
Supplement: Supplementary file 1 — Supporting Information [file ADVS-10-2303291-s001.pdf]

ADVANCED SCIENCE
Open Access

Supporting Information

for *Adv. Sci.*, DOI 10.1002/advs.202303291

Lepr-Expressing PDLSCs Contribute to Periodontal Homeostasis and Respond to Mechanical Force by Piezo1

Danting Zhang, Weimin Lin, Shuang Jiang, Peng Deng, Linfeng Liu, Qian Wang, Rui Sheng, Hui Sophie Shu, Lijun Wang, Weiguo Zou, Bo O. Zhou, Junjun Jing, Ling Ye, Bo Yu, Shiwen Zhang and Quan Yuan**

Supporting Information

Lepr-expressing PDLSCs contribute to periodontal homeostasis and respond to mechanical force by Piezo1

Danting Zhang, Weimin Lin, Shuang Jiang, Peng Deng, Linfeng Liu, Qian Wang, Rui Sheng, Hui Sophie Shu, Lijun Wang, Weiguo Zou, Bo O. Zhou, Junjun Jing, Ling Ye, Bo Yu, Shiwen Zhang, Quan Yuan**

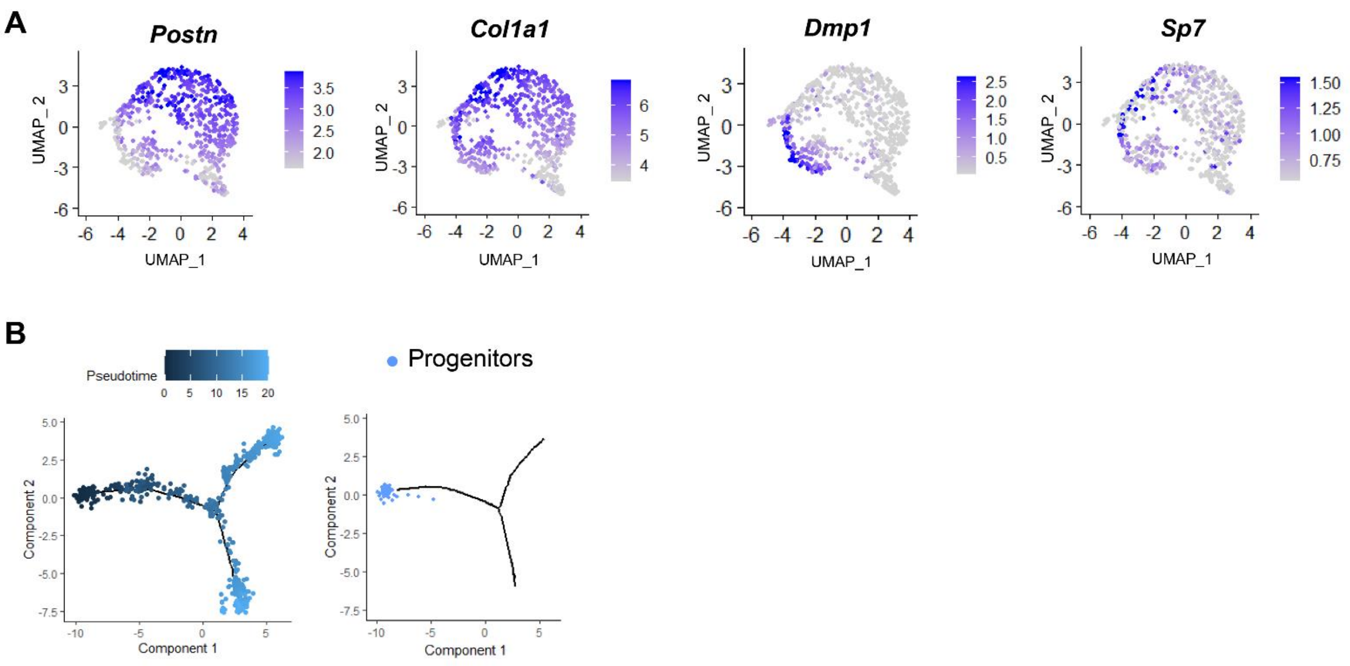

Supplementary Figure 1. Single-cell analysis of PDL stromal cells. Related to Figure 1.

A. The expression of marker genes projected onto UMAP atlas.

B. Left: The pseudotime developmental tree of PDL stromal cells. Right: Distribution of progenitors on the tree.

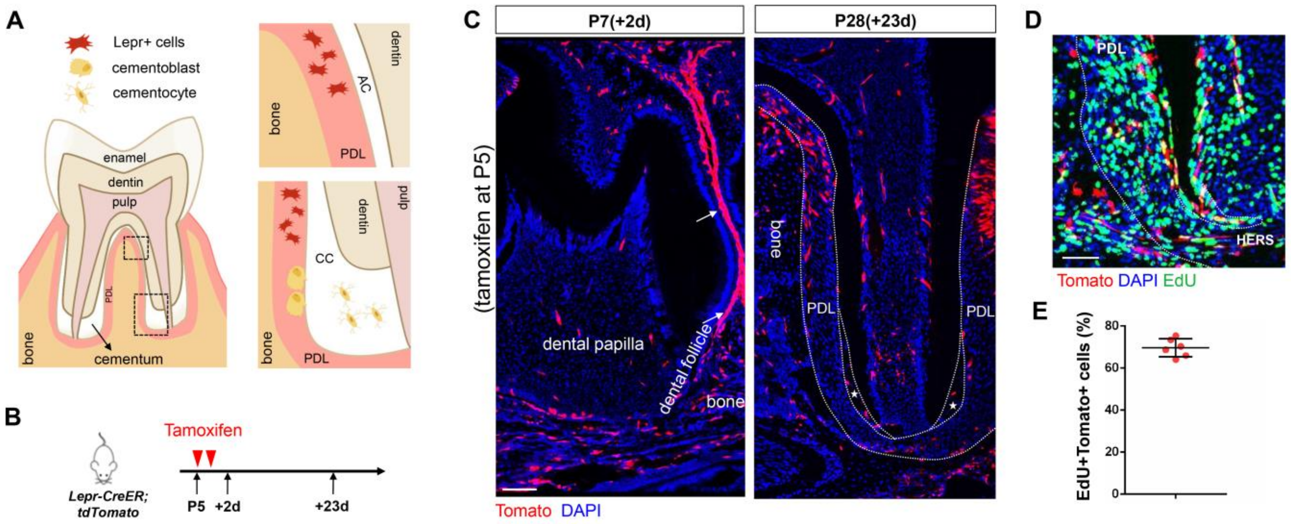

Supplementary Figure 2. Anatomy diagram and lineage tracing of Lepr^{+} cells from P5. Related to Figure 2.

A. Anatomy diagram of periodontal tissues. AC: Acellular cementum; CC: Cellular cementum.

B. Schematics of tamoxifen induction.

C. Representative confocal images of dental follicle and PDL from P5-induced *Lepr-CreER;tdTomato* mice. Scale bar: 100μm.

D-E. Representative EdU administration images and percentage of EdU+ Tomato+ cells/ Tomato+ cells from P5-induced *Lepr-CreER;tdTomato* mice at P14 (n=6). Scale bar: 50μm.

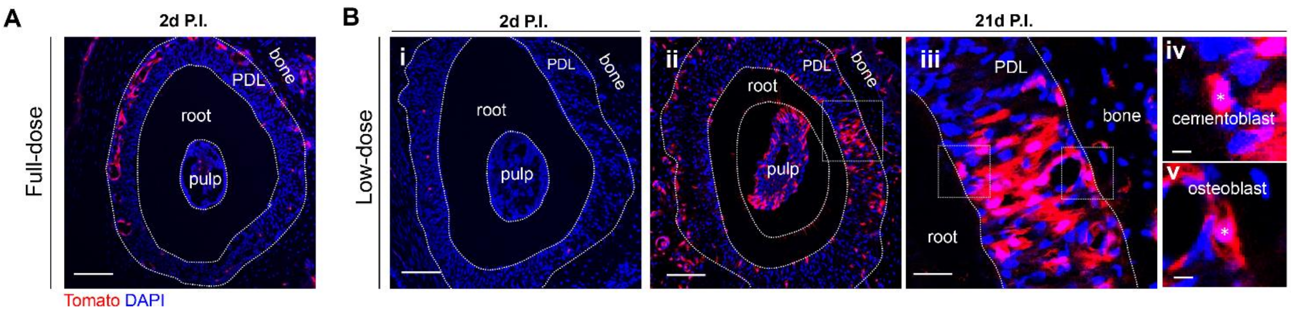

Supplementary Figure 3. Lepr+ cells clonal tracing *in vivo*. Related to Figure 3. *Lepr-CreER;tdTomato* mice were induced with various tamoxifen dose at the age of P21. (A) Regular full-dose (0.05 mg/g) for 2 days. (B) Single low(1/30)-dose (1.67µg/g). Images were shown on horizontal sections of the root.

A. Representative image of full-dose tamoxifen administration on *Lepr-CreER;tdTomato* mice which induced 2 days from P21. P.I., post-induction. Scale bar: 100µm.

B. Representative images of single low-dose tamoxifen administration on *Lepr-CreER;tdTomato* mice at the age of P21. Mice were sacrificed 2 days (i) or 21 days (ii-v) after induction. P.I., post-induction. Boxed regions in (iii) were enlarged to show Tomato+ osteoblast (iv) lining the alveolar bone or cementoblast (v) on the root surface. (i,ii) Scale bar: 100µm. (iii) Scale bar: 25µm. (iv,v) Scale bar: 5µm.

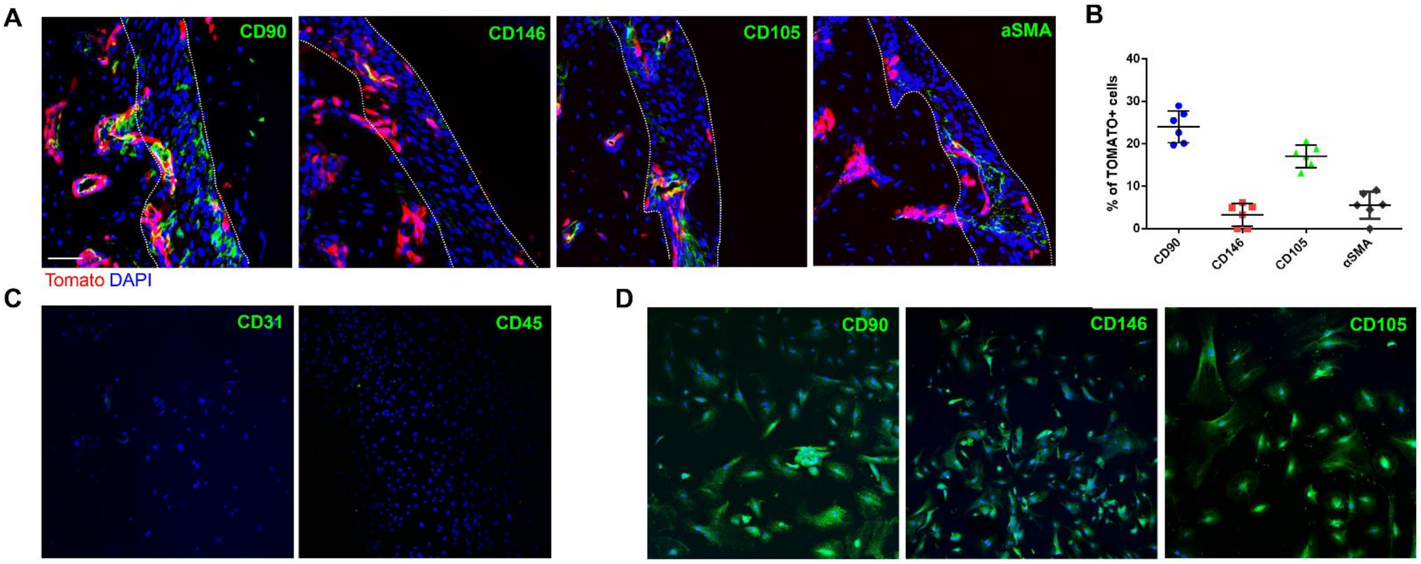

Supplementary Figure 4. Immunofluorescent staining *in vivo* and *in vitro.* Related to Figure 3.

A. Representative confocal images of immunofluorescent stem cell markers staining on adult *Lepr-CreER;tdTomato* mice. Scale bar: 50μm.

B. Percentages of positively stained cells/Tomato+ cells (n = 6).

C. CD31 and CD45 staining of PDLSCs *in vitro*.

D. CD90, CD146 and CD105 staining of PDLSCs *in vitro*.

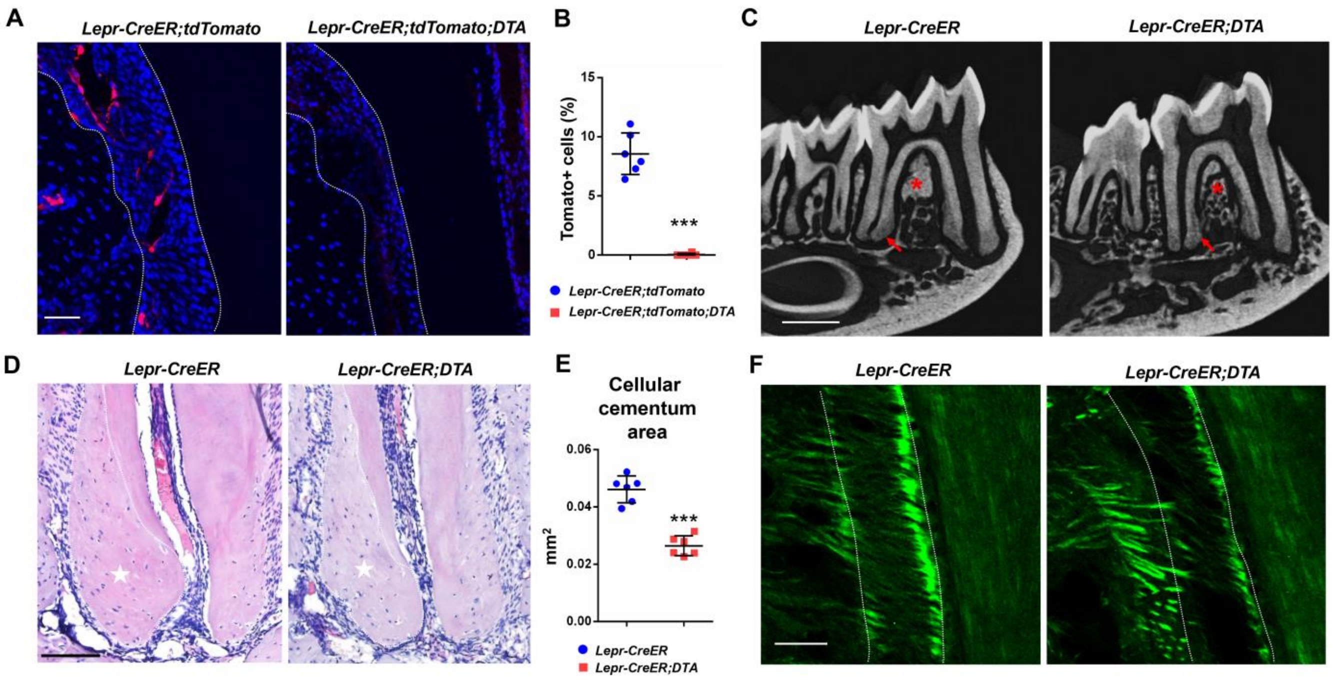

Supplementary Figure 5. Ablation of Lepr+ PDLSCs on adults disrupts periodontium homeostasis. Related to Figure 4.

A-B. Representative images and percentage of Tomato+ cells in PDL from adult *Lepr-CreER;tdTomato;DTA* and control mice (*n* = 6). Scale bar: 50μm.

C. Representative μCT images of mandible in *Lepr-CreER;DTA* and control mice. Scale bar: 500μm. Arrows indicate the cellular cementum. Asterisks indicate the alveolar bone.

D-E. H&E staining and statistical analysis of the cellular cementum in *Lepr-CreER;DTA* and control mice (*n* = 6). Scale bar: 100μm. Dotted lines outline ROI of the cellular cementum.

F. SHG microscopy of PDL fibrils in adult *Lepr-CreER;DTA* and control mice. Scale bar: 50μm.

Data are presented as mean ± standard deviation. ****P*<0.001 as determined by two-tailed Student t tests.

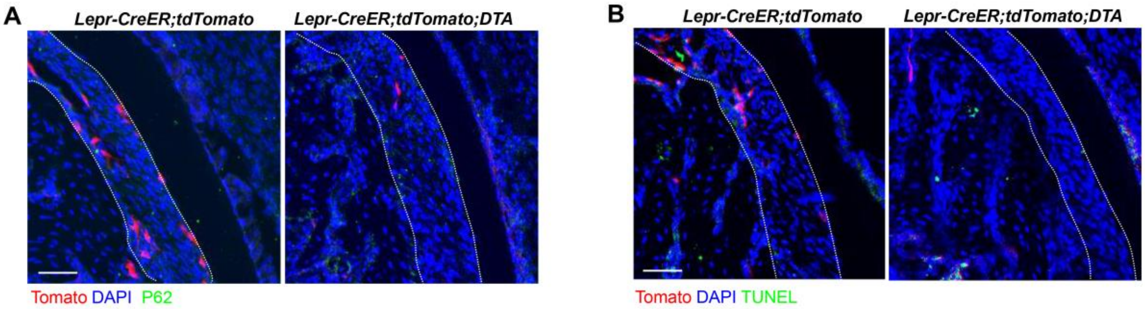

Supplementary Figure 6. Apoptosis and autophagy are not responsible for the disruption of PDL homeostasis after Lepr+ cells ablation. Related to Figure 4.

A. Representative images of P62 staining on *Lepr-CreER;tdTomato;DTA* and control mice. Scale bar: 50μm.

B. Representative images of TUNEL staining on *Lepr-CreER;tdTomato;DTA* and control mice. Scale bar: 50μm.

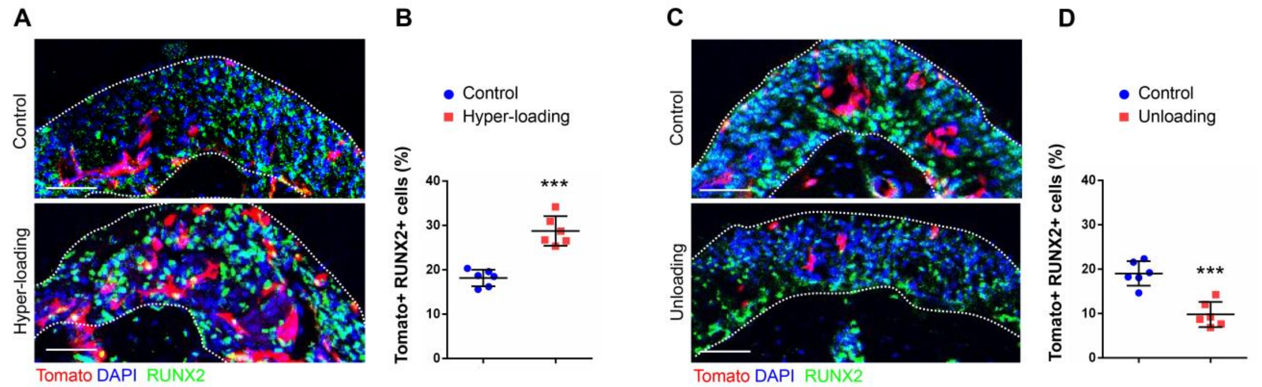

Supplementary Figure 7. Occlusal forces impact on osteogenic differentiation of Lepr+ PDLSCs. Related to Figure 5.

A-B. RUNX2 staining and percentage of Tomato+ RUNX2+/ Tomato+ cells on hyper-loading and control nice. (*n* = 6). Scale bar: 50μm.

C-D. RUNX2 staining and percentage of Tomato+ RUNX2+/ Tomato+ cells on unloading and control nice. (*n* = 6). Scale bar: 50μm.

Data are presented as mean ± standard deviation. ***P<0.001 as determined by two-tailed Student t tests.
